# Different Strategies for the Microfluidic Purification of Antibiotics from Food: A Comparative Study

**DOI:** 10.3390/bios13030325

**Published:** 2023-02-27

**Authors:** Lorenzo Lunelli, Martina Germanis, Lia Vanzetti, Cristina Potrich

**Affiliations:** 1 Bruno Kessler Foundation, Center for Sensors & Devices, Via Sommarive 18, 38123 Trento, Italy; 2 National Research Council, Institute of Biophysics, Via alla Cascata 56/C, 38123 Trento, Italy; 3 FTH Srl (Femtorays), Via Solteri 38, 38121 Trento, Italy

**Keywords:** antibiotic extraction, aptamer functionalization, antibody functionalization, microfluidic purification

## Abstract

The presence of residual antibiotics in food is increasingly emerging as a worrying risk for human health both for the possible direct toxicity and for the development of antibiotic-resistant bacteria. In the context of food safety, new methods based on microfluidics could offer better performance, providing improved rapidity, portability and sustainability, being more cost effective and easy to use. Here, a microfluidic method based on the use of magnetic microbeads specifically functionalized and inserted in polymeric microchambers is proposed. The microbeads are functionalized either with aptamers, antibodies or small functional groups able to interact with specific antibiotics. The setup of these different strategies as well as the performance of the different functionalizations are carefully evaluated and compared. The most promising results are obtained employing the functionalization with aptamers, which are able not only to capture and release almost all tetracycline present in the initial sample but also to deliver an enriched and simplified solution of antibiotic. These solutions of purified antibiotics are particularly suitable for further analyses, for example, with innovative methods, such as label-free detection. On the contrary, the on-chip process based on antibodies could capture only partially the antibiotics, as well as the protocol based on beads functionalized with small groups specific for sulfonamides. Therefore, the on-chip purification with aptamers combined with new portable detection systems opens new possibilities for the development of sensors in the field of food safety.

## 1. Introduction

Antibiotics are widely used both to treat and prevent infections in humans and animals, and to obtain favorable effects on animal growth. This widespread use has unwanted side effects, i.e., the possible contamination of the environment and of the food chain [1,2], with, for example, milk and meat for human consumption that may contain antibiotics [3,4,5]. In addition, the overuse of antibiotics adversely impacts the onset of antibiotic-resistant bacterial strains [6,7]. To minimize these problems, legislative bodies limit the amount of antibiotics that may be found in food for human consumption [8,9,10]. Besides standard laboratory technologies, several kind of sensors have been developed and commercialized to comply with these limits, with the aim of their potential use in small laboratories. These sensors are mainly based on lateral flow technologies or take advantage of ELISA (enzyme-linked immunosorbent assay) based methods. All these methodologies, however, suffer from the drawback of assessing the compliance of the raw food after its collection, implying that contamination may be spread to several batches of food during its processing, before the contamination may be detected. Ideally, raw food should be tested in the field at the beginning of the collection chain, leading to the isolation of contaminated batches from the following processing chain.

In this scenario, the development of automated purification methods may lead to the establishment of in-field usable instruments [11,12], which also allow automated data communication with centralized facilities. Using purification methods, traces of antibiotics could be separated from complex food matrices, easing their further analysis with automated methods [13]. This requires a two-step process, i.e., a first phase, where antibiotics are bound and the food matrix is removed, and a second phase, where the release of the captured antibiotics is implemented, using a medium that is far more simple than the original matrix. Several different capture strategies can be exploited, based on molecular-imprinted polymers [14], antibodies [15,16], aptamers [17,18,19] and also taking advantage of specific interactions that the molecules of interest have with small functional groups [20].

The ideal biosensor for the on-site detection of food contaminations should be able to process raw food and precisely quantify the possible presence of antibiotics. These characteristics are difficult to obtain in a single system since modern detectors are often based on label-free methods, such as SPR and electrochemical biosensors. SPR is highly sensitive, but the equipment is costly and not portable. On the contrary, electrochemical biosensors are not sensitive enough to apply them to real samples, due to the problem of interface effect on the electrode surface, which produces an extremely high background that impedes the proper quantification of biomolecules [21,22]. Many attempts to overcome these problems and to produce biosensors usable in real settings are based on nanomaterials [23,24,25], which, however, need a critical implementation toward the desired application. A combination among nanomaterials, microfluidics and new sensors could, therefore, increase dramatically the success of developing antibiotic biosensors for real sample detection [12,26]. With the aim of setting up a microfluidic purification system able to deliver simplified solutions to an innovative detector, such as the label-free sensors, here, different functionalization strategies were applied to microbeads inserted in a microfluidic device.

In this work, we focus indeed on the evaluation and comparison of the performance in terms of the efficiency of antibiotics’ capture and release, and of several binding strategies, namely aptamers, antibodies and small functional groups, using functionalized magnetic microbeads as a common platform. Two different kind of microbeads are evaluated, namely polystyrene based (Dynabeads${}^{\mathrm{TM}}$) and agarose based (PureCube), carrying different functional groups, with respect to their performance regarding the purification of the antibiotics tetracycline, sulfonamides and chloramphenicol.

## 2. Materials and Methods

### 2.1. Materials

The following reagents were purchased from Merck Life Science S.r.l. (Milan, Italy): methanol, acetonitrile, oxalic acid, ethanolamine, glycine, dithiothreitol (DTT), sulfanilic acid, hydrochloric acid, ammonium hydroxide, ammonium sulphate and all powders for buffer solutions.

The following microbeads were used: Dynabeads${}^{\mathrm{TM}}$ M-270 Epoxy (DynaEpoxy) and Dynabeads${}^{\mathrm{TM}}$ M-270 Streptavidin (DynaSA) from Thermo Scientific (Waltham, MA, USA); MAGAR-cN (MagarNA) from Immagina Biotechnology (Trento, Italy); PureCube NHS-Activated MagBeads (PureCubeNHS) and PureCube Maleimide-Activated MagBeads (PureCube Maleimide) from Cube Biotech (Monheim am Rhein, Germany).

All aptamers were obtained from IDT Integrated DNA Technologies (Leuven, Belgium). Aptamers are listed in Table 1 and Table 2.

Anti-antibiotics antibodies were obtained from Fitzgerald (Fitzgerald Industries International; Acton, MA, USA): sheep polyclonal anti-tetracycline antibody (a-TC Ab); sheep polyclonal anti-chloramphenicol antibody (a-CAF Ab); and rabbit polyclonal anti-sulfamethazine antibody (a-SMZ Ab). The fluorescent antibody Ab-A568 (rabbit anti-goat IgG (H+L), Cross-Adsorbed Secondary Antibody, AlexaFluor${}^{\mathrm{TM}}$568 and protein G were purchased from Thermo Scientific (Waltham, MA, USA).

The following buffers were used:

Binding Buffer (BB; used for TC and SMZ): 100 mM NaCl, 2 mM MgCl_2_, 5 mM KCl, 1 mM CaCl_2_, 20 mM Tris/HCl pH 7.6

Binding Buffer Muller (BBM): 100 mM NaCl, 10 mM MgCl_2_, 20 mM potassium phosphate buffer pH 7.5

Binding and Washing Buffer (B&W): 1 M NaCl, 0.5 mM EDTA, 5 mM Tris/HCl pH 7.5

Coupling Buffer I (CBI): 150 mM NaCl, 100 mM sodium phosphate pH 7.4

Phosphate Buffered Saline (PBS): 138 mM NaCl, 2.7 mM KCl, 10 mM sodium phosphate pH 7.4

Binding Buffer SDM (BBSDM): 50 mM NaCl, 5 mM KCl, 5 mM MgCl_2_, 20 mM Tris/HCl pH 8

The Istituto Zooprofilattico Sperimentale del Piemonte, Liguria e Valle d’Aosta (IZSPLV), provided raw milk and honey, all tested for the absence of antibiotics. IZSPLV also provided pure tetracycline, sulfonamides and chloramphenicol for spiking solutions.

Microfluidic chips, with four chambers of 100 µL volume each (Rhombic Chamber Chip eP1, Fluidic Design 221, PMMA, 600 µm depth), were purchased from microfluidic ChipShop GmbH (Jena, Germany).

### 2.2. Interaction of Antibiotics with Their Aptamers or Antibodies

The specific recognition of aptamers and antibodies (in this section generally designed as macromolecules) with their targets was evaluated in solution, using two different methods: the effect of binding on the intrinsic fluorescence of tetracycline was exploited for testing the a-TC aptamers [17] (Section 2.2.1), while an equilibrium filtration method [27,28] was utilized for all aptamers (apart from a-TC8, which is too small for this method), and for all the antibodies (Section 2.2.2).

#### 2.2.1. Spectrofluorimetric Analysis

Different concentrations of TC were tested for their intrinsic fluorescence signal in order to optimize the concentration to be used as starting amount. Therefore, TC was dissolved at fixed nanomolar concentration in a total volume of 1 mL of buffer 100 mM NaCl, 2 mM MgCl_2_, 5 mM KCl, 1 mM CaCl_2_, 20 mM sodium phosphate pH 7.6 for a-TC8, a-TC40 and a-TC76 or BBM for a-TCmu and specific aptamers were added at increasing concentrations. The aptamers were titrated in such a way as to not exceed a total volume increase of 5%. The solution was stirred during each titration step and allowed to equilibrate for 5 min before data collection (longer incubation time were also tested, without significant differences in results). Fluorescence spectra were acquired for each titration point with a SPEX FluorMax spectrofluorimeter (Horiba Instruments Inc., Edison, NJ, USA) at 25 °C. An excitation wavelength of 370 nm was used to acquire the emission spectrum from 380 to 610 nm. The fluorescence emission signal from 500 to 535 nm was then integrated and plotted against the aptamer concentration.

#### 2.2.2. Equilibrium Filtration Method

Vials with 100 µL solutions of macromolecules:antibiotic (1:1 volumes) were prepared at a fixed antibiotic concentration and increasing macromolecule concentration and left to reach equilibrium for 30 min. Afterwards, the solutions were transferred in the upper compartment of filters with a nominal cutoff of 10.000 Dalton (Microcon-10kDa Centrifugal Filter Unit with Ultracel-10 membrane, Merck Life Science S.r.l.; Milan, Italy), or 3.000 Dalton (Amicon Ultra-0.5 Centrifugal Filter Unit, Ultracel-3, Merck Life Science S.r.l.; Milan, Italy), depending on the MW of the tested aptamer. Then, the solutions were centrifuged for 8 min at 12.000× *g*, allowing around 50–60 µL of solution to flow to the lower filter compartment. The antibiotic concentration found in the flow through is a good estimate of the free antibiotic present in the original reaction vial. On the other hand, it is expected that the antibiotic bound to the aptamer is (mostly, see below for details) confined in the upper filter compartment.

#### 2.2.3. Fit of the Antibiotic-Macromolecule Titration

The amount of bound antibiotic present in the solutions prepared as described above, was estimated from the saturation parameter ν≡L/M, i.e., defined as the moles of bound ligand (antibiotic) per mole of macromolecule (aptamer or antibody), expressed using the equation from Cantor and Schimmel Biophysical chemistry (section 15-3, [29]). The parameters obtained from this equation were used to build two different models for TC fluorescence and equilibrium filtration experiments. The fitting of experimental data was implemented in Octave v. 6.1.0 [30], with the optim package v.1.6.0, using TeXmacs v. 2.1.1 [31] as the graphical front-end. The detailed description of the fitting method as well as the definition of the fitting parameters is detailed in the Supplementary Materials file. In brief, the equilibrium constant *k* is obtained in both models, while the fluorescence yields for the free ($F_{f}$) and bound ($F_{b}$) TC characterize the fit of fluorescence experiments.

Concerning the fit of equilibrium filtration experiments, the fitted parameters are (alongside *k*) the filter throughput losses λ and the aspecific antibiotic adhesion ($TC_{aspecific}$ —for TC).

### 2.3. Functionalization of Microbeads

The microbeads were functionalized by adapting the manufacturer’s instructions, as detailed in the Supplementary Materials file.

*DynaEpoxy* and *PureCubeNHS*. These beads were conjugated either to amino-terminated aptamers or to the primary amines exposed by the basic amino acids in antibodies or in protein G, used as an intermediate step to better orient the antibody molecules. Finally, DynaEpoxy was conjugated also with sulfanilic acid for the capture of sulfonamides, adapting the protocol described by Hirsch et al. [32]. All fractions were collected and measured at the spectrophotometer in order to quantify the bound aptamer/antibody/sulphone groups (Section 2.4.3).

*DynaSA* and *MagarNA.* These beads expose streptavidin on their surface and were made to react with biotinilated aptamers. Additionally, in this case, the supernatant collected after reaction was measured, and the amount of bound aptamer was quantified (Section 2.4.3).

*PureCube Maleimide*. Thiolated aptamers, reduced just before use, were conjugated to these beads, as detailed in the Supplementary Materials

### 2.4. Characterization of the Functionalized Microbeads

The binding of aptamers or antibodies to the beads was monitored by binding a fluorescent aptamer or antibody to the beads and checking the resulting fluorescence via confocal microscopy. Moreover, all the solutions of aptamer/antibody used for the functionalization of the microbeads were collected and quantified with the spectrophotometer to indirectly estimate the amount of bound aptamer/antibody. When PureCubeNHS beads are functionalized, this spectrophotometric determination of the bound aptamer/antibody is impossible because during the procedure, the NHS group is released, whose absorbance maximum is close to that of aptamers and antibodies.

In addition, the XPS analysis was employed for monitoring the functionalization of beads.

#### 2.4.1. Confocal Analysis

The microbeads functionalized with the fluorescent aptamer or antibody were resuspended in PBS, deposited on a microscope slide and covered with a coverslip. A Leica SP5-II confocal microscope (Leica Instruments, Wetzlar, Germany), equipped with a helium/neon laser (543 nm), was employed for imaging the beads, acquiring data with a 40× objective in air. Images were also acquired using the additional channel of the transmitted light photomultiplier to image all the microbeads, independently of their functionalization. Spectral data of functionalized and untreated microbeads were acquired to confirm the correspondence of the detected light with the fluorescence spectra of the used fluorophores.

#### 2.4.2. XPS Analysis

The XPS analysis is particularly suitable for the chemical characterization of samples carrying biomolecule layers, as already shown [33]. Samples for XPS measurements were deposited as 15 µL solutions of microbeads resuspended in pure water, deposited on 1 cm × 1 cm substrates of thermally grown silicon oxide, after improving the beads adhesion with argon plasma treatment (10.5 W, 2 mbar, 1 min). Samples were carefully dried at room temperature before their introduction in the chamber. XPS analyses were performed using a Kratos Axis Ultra${}^{DLD}$ instrument equipped with a hemispherical analyzer and a monochromatic AlKα (1486.6 eV) X-ray source, in spectroscopy mode.For beads functionalized with sulfanilic acid, O 1s, C 1s, N 1s, S 2p core lines were acquired, while for beads functionalized with a-TC Ab, O 1s, C 1s, N 1s, and Si 2p core lines were measured. XPS quantification was performed using the instrument sensitivity factors and the high-resolution spectra. Charge compensation was achieved using a charge neutralizer located at the bottom of the electrostatic input lens system. The quantification, reported as a relative elemental percentage, was performed by using the integrated area of the fitted core lines and by correcting for the atomic sensitivity factors. XPS data analysis was performed using the software described in Speranza and Canteri [34].

#### 2.4.3. Spectrophotometric Analysis

All solutions produced during the functionalization of microbeads as well as the stock solutions added to the beads were collected and measured at the spectrophotometer (Jasco V-550) to check the amount of aptamer/antibody present. Spectra from 190 to 340 nm were acquired and the absorbance at 260 (aptamers) or 280 nm (antibodies) was used to quantify the amount of aptamers (considering A_260_ ssDNA = 33 µg/mL) and antibodies (considering an approximate $\varepsilon_{280}$IgG = 210,000).

### 2.5. Binding/Elution Test

The binding of antibiotics to their specific targets, which are bound to microbeads, was studied both in 1.5 mL vials and using fluidic microdevices. The test in vials started from the functionalized microbeads, which are incubated with the antibiotics at fixed concentrations for 1 h at room temperature using a tilting and rotation mixer. At the end of incubation, the vials were mounted on a magnet, the supernatant was collected (unbound antibiotic), and the microbeads were washed for at least three times with the suitable binding buffer. Then, different conditions of ionic strength, pH, and temperature were tested for the elution of the antibiotics. A final washing step was then performed. All fractions were collected for quantification in HPLC.

**Figure 1:**
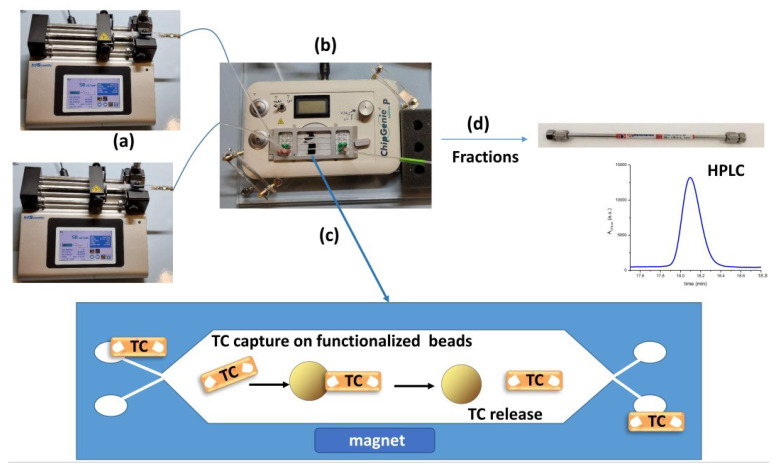
Scheme of the microfluidic purification of antibiotics. Two syringe pumps (**a**) inject the fluids into a microfluidic chip positioned on a device (**b**) equipped with a magnet. The chip, previously filled with the functionalized magnetic microbeads, is fluxed with a solution containing the antibiotics (TC; step (**c**)), which are captured by the beads. When a suitable elution solution is fluxed, the antibiotics are released from the beads and collected for analysis with HPLC (**d**).

The binding test performed on the microfluidic chip was adapted from Lunelli et al. [20] and is schematized in Figure 1. Briefly, a microfluidic PMMA chip was mounted on the ChipGenie ®edition P device (microfluidic ChipShop, Jena, Germany; in Figure 1b) and fluids were injected in the chip chamber inlets with two disposable syringes actuated by two syringe pumps (Legato 185, KD Scientific, Holliston, MA, USA; in Figure 1a). The chip chambers were filled with functionalized magnetic beads at the beginning of the experiment (typically 30 µL diluted in 350 µL of suitable buffer), and the solutions containing known amounts of antibiotic were loaded in the microdevice (step in Figure 1c). The antibiotic is captured on the beads’ surfaces, while all the unwanted materials present in the raw matrix are washed away with the buffer. The antibiotic is then released with the proper elution condition (high ionic strength solutions, extreme pH, and temperature). Finally, a washing step with buffer is performed. All fractions were collected from the outlet for the quantification via HPLC (step in Figure 1d). Concerning the milk experiment, no preliminary treatments or dilution in the binding buffer was performed before the spiking of TC. Honey instead required a preliminary dilution of 5 times in the binding buffer.

### 2.6. Quantification of Antibiotic Solutions

The antibiotic present in the fractions collected during the binding/elution test (both in 1.5 mL vial and microdevice) was quantified by means of a Shimadzu (Kyoto, Japan) HPLC instrument. The instrument was equipped with the UV/Vis Detector SPD-20A, the Shimadzu prominence Communications Bus Module CBM-20A, the binary pump system Liquid Chromatograph LC- 20AB and with a degasser (Waters${}^{\mathrm{TM}}$ In-Line Degasser) to improve HPLC performance. Fractions, after centrifugation at 21,000× *g* for 10 min, were manually injected through a Rheodyne® model 7725i injector connected with a 50 µL loop and passed through a precolumn Security Guard${}^{\mathrm{TM}}$ by Phenomenex® before separation by means of an inverse phase column (Luna® C18 by Phenomenex®, 5 µm, 10 nm, 250 mm × 3 mm), acquiring data with the software provided with the instrument (LCSolution). The mobile phase for tetracycline was oxalic acid 0.01 M, pH 2.7 (solvent A), and the gradient was obtained with methanol and acetonitrile in a 1.5:1 ratio (solvent B). Flux was set to 0.35 mL/min. The separation method was the following: 10% solvent B for 5 min, and gradient up to 58% of solvent B in 20 min. For the quantification of sulfonamides, 0.05 M sodium acetate pH 4.8 was used as a mobile phase (solvent A) and pure acetonitrile as solvent B. The separation method was gradient from 20% of solvent B to 50% B in 23 min, and 1 min up to 70% B. For the quantification of chloramphenicol, 0.1% formic acid in water was used as solvent A and 0.1% formic acid in acetonitrile as solvent B, while the separation method was 20% of solvent B for 5 min, gradient up to 95% B in 15 min. A calibration curve was obtained for each antibiotic by injecting known amounts of antibiotic in HPLC; the fit of this curve allowed the precise quantification of the antibiotic present in each fraction.

## 3. Results and Discussion

**Figure 2:**
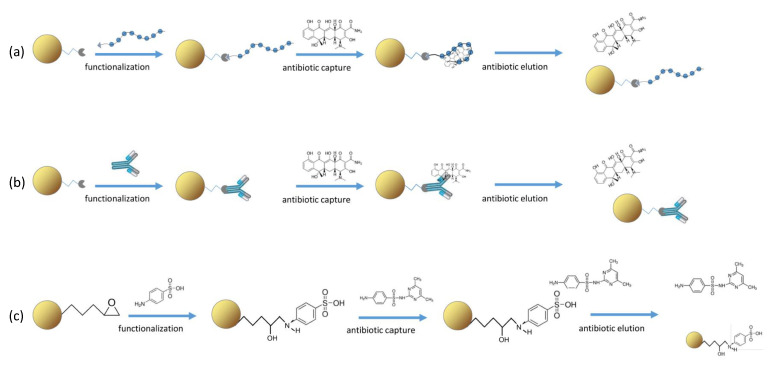
Strategies for the purification of antibiotics. Beads exposing specific functional groups are covalently coupled to the recognition element (functionalization), which binds its antibiotic target (antibiotic capture). Molecules present in the matrix are washed away, while the antibiotic is retained. By changing the environmental conditions (pH, ionic strength, temperature), the antibiotic is released (antibiotic elution) and collected for further analyses. (**a**) Strategy based on beads functionalized with aptamers as recognition element; (**b**) strategy based on beads functionalized with antibodies as recognition element; (**c**) strategy based on beads functionalized with sulfone groups for the purification of sulfonamides.

Different strategies implementable on microfluidic devices were tested, and their performance in terms of antibiotics capture and release were compared. All strategies are based on the use of microbeads exposing different functional groups, which were exploited for the specific functionalization either with aptamers (Figure 2, strategy a), or antibodies (Figure 2, strategy b), or small molecules such as sulfanilic acid (Figure 2, strategy c). The morphology of all the tested microbeads was checked by field-emission scanning electron microscopy before functionalization (Figure S1 of Supplementary Materials). The functionalization of beads was carefully monitored as well as the interaction of aptamers/antibodies with their specific targets before using the functionalized beads for the on-chip purification of antibiotics.

### 3.1. Strategy Based on Beads Functionalized with Aptamers

The first strategy explored for the microfluidic purification of antibiotics possibly present as contaminants in food, is based on the specific interaction of aptamers with their target antibiotics. Many aptamers are reported in the literature as specific for antibiotics, in particular, for TC. Here, four aptamers recognizing TC were selected and tested (see Table 1): a-TC76, a DNA-aptamer 76 nucleotides long and two shorter DNA aptamers, a-TC40 and a-TC8, partially sharing the same sequence as a-TC76 [35,36,37]. A fourth aptamer based on a RNA sequence was also tested [17]. In addition, two aptamers specific for sulfonamides were analyzed, i.e., a-SDM [38] and a-SMZ [18,39].

#### 3.1.1. Aptamers–Antibiotics-Free Interaction in Solution

(a) Fluorescence test

**Figure 3:**
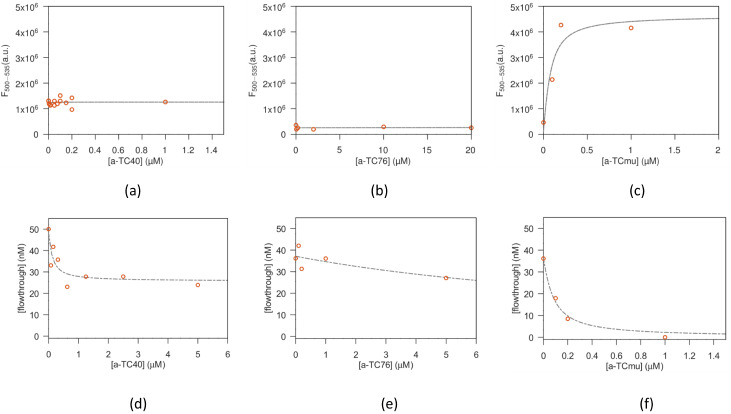
Binding tests of tetracycline and aptamers: circles (experimental data), dashed lines (fit). Fluorescence emission data for TC incubated with aptamer a-TC40 (**a**), a-TC76 (**b**) and for a-TCmu (**c**). Data obtained with a-TC8 are similar to (**b**). Data obtained with the filtration test for tetracycline and a-TC40 aptamer (**d**), a-TC76 aptamer (**e**) and a-TCmu aptamer (**f**).

The binding affinity of anti-TC aptamers was tested in solution, exploiting the intrinsic fluorescence of TC [17], which may change when aptamers interact with the antibiotic. Starting from a fixed nanomolar amount of TC, increasing amounts of aptamers were added, and the fluorescence spectra were acquired. When the aptamer binds to TC, the fluorescence signal changes from $F_{f}\cdot TC$ and tends to $F_{b}\cdot TC$ at high aptamer concentration. This leads to an increase in fluorescence, if $F_{b}>F_{f}$. The experimental binding plots are reported in Figure 3a–c. As can be observed, only the a-TCmu aptamer leads to a measurable increase in fluorescence, obtaining in this case a value of ≃ 60 nM for the equilibrium constant *k*, and an increase of ≃ 12 times for the tetracycline fluorescence yield, when bound. When a-TC8 (data not shown), a-TC40 and a-TC76 are employed, instead, no measurable changes in fluorescence intensity are detected. Correspondingly, in these cases, $F_{b}≃F_{f}$ is obtained from the fit and no *k* values can be obtained for these aptamers with this method.

(b) Equilibrium filtration test

The anti-TC aptamers were then tested with the equilibrium filtration method (Figure 3d–f). Because of its very low MW (2830 dalton), well below the nominal filter cutoff of 10 kD and 3 kD, a-TC8 was not tested. The parameters obtained from the fitting procedure are summarized in Table 3. The value of *k* obtained for the a-TCmu aptamer compares very well with the value obtained with the fluorescence method. For the a-TC40 aptamer, a *k* of the same order of magnitude is obtained, while for the a-TC76 aptamer, a much lower binding is measured. Note that when the a-TC40 aptamer is employed, a noticeable filter leakage is detected, as expected considering the MW of this aptamer (13 kD), not far from the filter cutoff of 10 kD.

**Figure 4:**
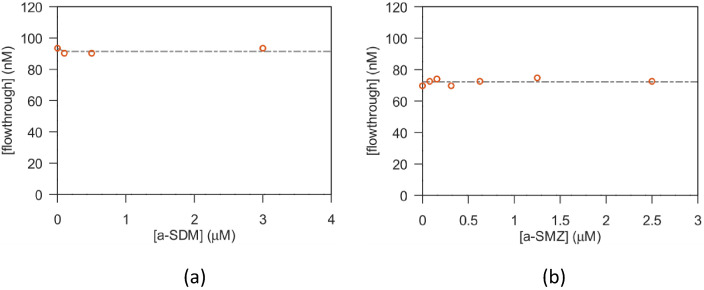
Filtration test of sulfonamides. (**a**) Data obtained with sulfadimethoxine and a-SDM aptamer. (**b**) Data obtained with sulfamethazine and a-SMZ aptamer. Y axes show the concentration of antibiotic present in the filter flow through.

The same test was performed on SMZ and SDM sulfonamides, using a-SMZ and a-SDM aptamers, respectively. Unfortunately, as shown by the plots reported in Figure 4, no effect of binding was detected. In fact, the *k* value of a-SMZ results far higher than the range of explored aptamer concentration, i.e., larger than 2.5 µM. Therefore, in the tested conditions, no apparent interaction of SDM neither of SMZ with their specific aptamers was observed. To be noted, the filtration test with the a-SDM aptamer was performed with filters of 3 kD nominal cutoff since the MW of the a-SDM aptamer is approximately 7.4 kD, i.e., lower than the cutoff of the standard filters used for all other aptamers.

#### 3.1.2. Functionalization of Microbeads with Aptamers

**Figure 5:**
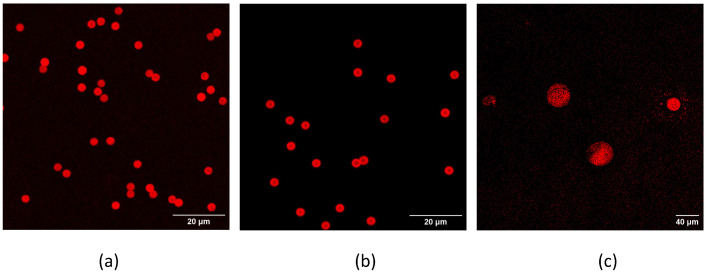
Confocal analysis of fluorescent aptamers bound to beads: DynaEpoxy beads conjugated with a-TC8 labeled with TAMRA (**a**), DynaSA conjugated with a-TC40 TAMRA (**b**), and PureCube Maleimide with a-TC76 TAMRA. Scale bars represent 20 µm in panels (**a**,**b**), while in (**c**) is referred to 40 µm.

The ideal conditions for the interaction aptamer target could be very different when this couple is present in a solution with respect to having the aptamer bound to the microbeads surface. For this reason, aptamers were coupled to beads different for the dimensions, material and exposed chemical group (for reference, see [40]). Amino-terminated aptamers were covalently conjugated to beads exposing epoxy or NHS groups, while thiolated aptamers were covalently conjugated to maleimide beads and biotinilated aptamers to beads coated with streptavidin or neutravidin. The binding was verified with a spectrophotometer, when possible, and by confocal analysis, using labeled aptamers. Figure 5 shows some examples of beads carrying different chemistry (epoxy–amino in (a), streptavidin–biotin in (b) and maleimide–thiol conjugation in (c)), after their functionalization with aptamers. For the confocal analysis, all aptamers employed were synthesized with a suitable reactive group at 5${}^{\prime }$ end and a fluorophore at the 3${}^{\prime }$ end. All types of chemistry worked well concerning the binding of the aptamers, independently of the beads dimensions (in the range from few µm to tens of µm) and materials (polymers vs. agarose). After aptamer binding, all beads were fluorescent (by comparison with pictures of the same area, acquired using transmitted light, data not shown), indicating a good yield of the aptamer conjugation.

#### 3.1.3. Capture and Release of Antibiotics from Functionalized Microbeads

**Figure 6:**
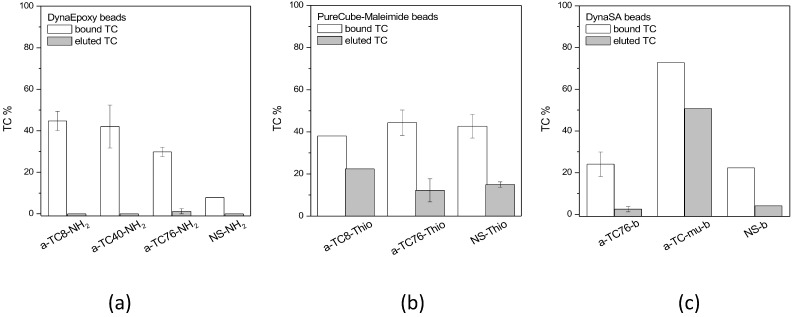
Binding of tetracycline to beads functionalized with aptamers. The percentage of TC is referred to the initial antibiotic added to the beads and set as 100% TC. (**a**) Beads exposing epoxy groups functionalized with amino-terminated aptamers; (**b**) beads exposing maleimide groups functionalized with thiol-terminated aptamers; (**c**) beads exposing streptavidin functionalized with biotin at 5′. The elution step was performed with 2 M NaCl and heating at 80 °C. All reactions were carried out in 1.5 mL vial, fractions of each step were collected and quantified with HPLC. NS: nonsense aptamers. Means and standard errors are reported.

Upon assessing the conjugation of aptamers to the microbeads, a binding test was setup, firstly in vial and then on-chip. The different microbeads functionalized with anti-antibiotic aptamers were incubated with the corresponding antibiotic and solutions were collected after each step of the test (unbound, washes, elution, and final wash). All the collected fractions were quantified via HPLC in order to estimate the amount of antibiotic captured and released from aptamer-functionalized beads. All the a-TC aptamers bound to DynaEpoxy beads captured a good amount of the target, significantly higher than the nonsense (NS) aptamer (Figure 6, panel a), but no elution was observed. However, when a different elution step, i.e., glycine 100 mM pH 2.3, was implemented for a-TC8 and a-TC40 bound to DynaEpoxy, the percentage of eluted TC was between 9 and 11%, and similar results were obtained with these two aptamers conjugated to PureCubeNHS beads.

Moving on to a-TC aptamers conjugated via thiol chemistry to PureCube Maleimide beads, they seem to capture a quite good amount of TC and also the eluted TC seems in good percentage (Figure 6, panel b). Unfortunately, the NS aptamers gave in this case similar performance results. Finally, among biotinylated aptamers bound to DynaSA beads a-TCmu stands out both for the capture and elution of TC (Figure 6, panel c). This RNA aptamer resulted then as the most promising aptamer for the on-chip purification of TC.

The anti-sulfonamides aptamers were also tested in similar conditions, obtaining a small capture of SMZ (around 2%) by the a-SMZ-b aptamer bound to DynaSA, while no capture of SDM could be measured with the same chemistry, i.e., a-SMZ-b and DynaSA (data not shown). Analogously, as expected, no elution was observed for both the tested sulfonamides. To be noted, both aptamers are conjugated to the beads, as verified with the spectrophotometric analysis. Nevertheless, these results are in good agreement with the data of filtration test (Figure 4).

**Figure 7:**
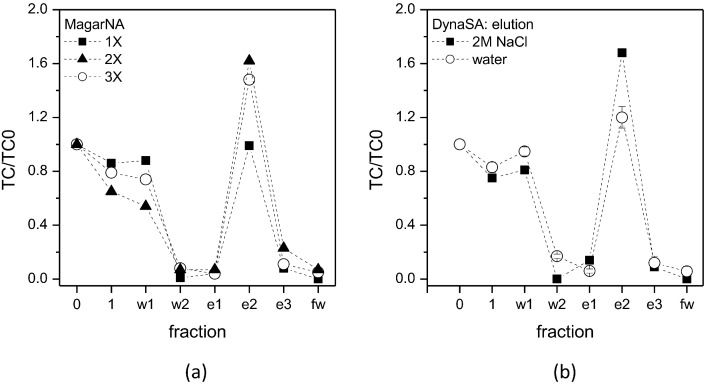
Microfluidic test. MagarNA beads (**a**) or DynaSA beads (**b**) were functionalized with a-TCmu aptamer and inserted in a 100 µL chip chamber before fluxing TC at 0.1 ng/µL concentration (fraction named 0). Consecutive fractions were collected and quantified with HPLC: fraction 1 represents unbound TC, fractions w1 and w2 are two successive washes, fractions e1-e3 represent eluted TC and fraction fw is the final wash. In (**a**) different amount of functionalized beads are tested, while in (**b**) different elution methods are shown, i.e., high ionic strength (2M NaCl) and high temperature (water at 75 °C).

The binding test performed in vials allowed to select the most promising aptamer for the implementation of the on-chip purification of antibiotics. The a-TCmu aptamer was selected for the purification of tetracycline, binding its biotinylated form to MagarNA or DynaSA beads. The functionalized beads were inserted in the chip chamber and TC was fluxed at the lowest TC concentration allowed in milk (maximum residue limit or MRL of 100 µg/kg or 0.1 ng/µL [8,9]), and then eluted with a high ionic strength solution or with high temperature in order to promote a structural change in the aptamer sequence, releasing TC. Fractions of the flowing solutions were collected during the experiment and quantified (Figure 7). Different amounts of functionalized MagarNA beads were tested (Figure 7, panel a), finding a higher TC recovery when a double amount of beads was used, while 3 times more beads did not further improve the purification performance results. Moreover, two different conditions were tested for TC elution, i.e., 2 M NaCl (high ionic strength) and high temperature (75 °C in water), as shown in Figure 7, panel b. In all the tested conditions, TC not only could be purified from the starting solution but was also concentrated up to two times in the eluted fractions. Interestingly, the elution in water is particularly promising since TC could be further concentrated by evaporating water without any contaminants. This result opens the way for a sensitive detection and analysis of pre-purified antibiotics with modern technologies, such as label-free methods. Moreover, the possibility to concentrate antibiotics with respect to the initial sample could be crucial for detecting minute traces of antibiotic residues.

Given the good results obtained starting from TC spiked in buffer (Figure 7), the on-chip purification was extended to TC in milk or honey. Unfortunately, 0.1 ng/µL of TC spiked in raw milk or in milk diluted with binding buffer up to 10 times gave no positive results, possibly because of the matrix effects on the capture of TC by a-TCmu. A similar protocol was tested for TC spiked in honey diluted 5 times in binding buffer, at 1 ng/µL concentration. In this case, a little amount of TC could be recovered (TC/TC0 for e2 fraction was 0.05; elution was performed at high temperature) but far from the results obtained for the on-chip purification of TC spiked in buffer. A possible explanation of this result is that honey contains many different compounds able to interfere with the aptamer–antibiotic binding [41,42]. Good binding results are indeed reported only when honey is treated to a great extent before analysis [43]. Similarly, analogous data were obtained when TC spiked in raw milk was processed on PureCubeNHS beads functionalized with a-TC8 aptamer (TC/TC0 for the eluted fraction e2). Additionally, milk contains several compounds that could interfere with the aptamer–antibiotic binding [44,45], while this adverse effect is less present when the interaction of antibiotics is mediated by small molecules or even ions [20].

### 3.2. Strategy Based on Beads Functionalized with Antibodies

Besides aptamers, antibodies recognizing antibiotics have been developed despite the difficulty given by the small size of the antibiotic molecules [46,47]. Antibodies or protein G were covalently conjugated to epoxy or NHS beads. When protein G is bound, the antibodies are specifically bound to protein G with their Fc region, obtaining an oriented layer of antibodies (see for details [40]). Three antibodies recognizing antibiotics belonging to three different classes were selected to implement the on-chip purification based on microbeads functionalized with a-TC Ab, a-SMZ Ab and a-CAF Ab, respectively (strategy b in Figure 2).

#### 3.2.1. Antibodies-Antibiotics Free Interaction in Solution

**Figure 8:**
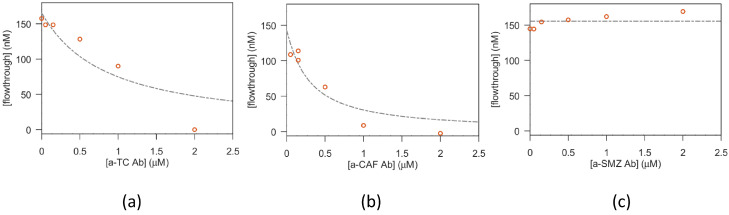
Characterization of binding between TC/CAF/SMZ and their Abs in solution (filtration test).

As reported for aptamers in Section 3.1.1, also the antibody–antibiotic interaction was evaluated in solution using the equilibrium filtration method. Figure 8 reports the data obtained for the antibiotics TC, CAF and SMZ. The corresponding values of *k* are reported in Table 4. While a detectable interaction was obtained for TC and CAF, again, as obtained with the a-SMZ aptamer, no measurable interaction was found for SMZ.

Since the interaction in solution or on the microbeads surface could give different results, the binding test was performed for all the three antibodies conjugated to the respective microbeads.

#### 3.2.2. Characterization of the Antibody Binding to the Microbeads

**Figure 9:**
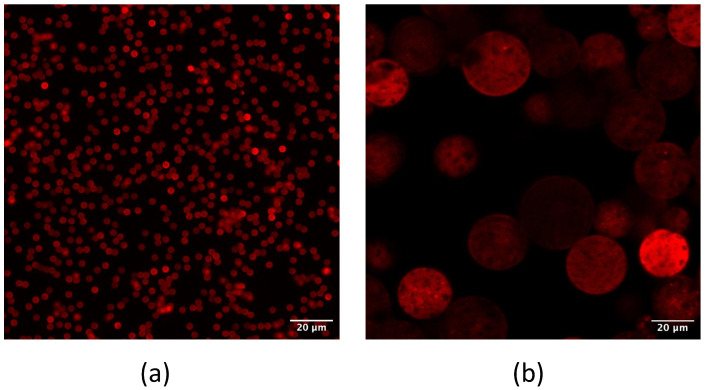
Characterization of the binding of a fluorescent antibody to beads (confocal analysis). Ab-A568 was conjugated either to DynaEpoxy beads coated with protein G (**a**), or to PureCubeNHS beads (**b**). Scale bars represent 20 µm.

Before testing the binding of antibiotics to the functionalized beads, the successful conjugation of antibodies to the surface of both DynaEpoxy and PureCubeNHS was verified with the confocal analysis. A fluorescent antibody (Ab-A568) was conjugated following the same protocol developed for DynaEpoxy beads (i.e., binding of ptG to epoxy groups through amino residues, followed by passivation with ethanolamine and incubation with the proper antibody; see Section 2.3) or for PureCubeNHS and then was measured with the confocal microscope. All beads were found to be coated with the fluorescent antibody, as clearly visible in Figure 9), and as resulted from the comparison of same areas acquired using transmitted light (data not shown).

For DynaEpoxy beads, a quantification of the amount of antibodies bound to the beads was possible via spectrophotometric analysis, while unfortunately this was not possible for PureCubeNHS, which released the NHS group during the conjugation process. The NHS group absorbs near the typical protein absorption band (i.e., 260–280 nm), giving as a result a broad peak comprising both phenomena. a-TC Ab was selected for the characterization of the binding to the DynaEpoxy beads, finding a percentage of bound antibody of (33 ± 15) % and (1.2 ± 0.3) $\times 10{}^{12}$ molecules of antibody/cm${}^{2}$ of beads surface, starting from the 0.3 mg/mL of antibody added during beads-Ab incubation.

The successful functionalization with antibody was checked for DynaEpoxy beads and a-TC Ab also with XPS measurements, acquiring the N 1s signal. DynaEpoxy indeed presents a small amount of nitrogen on its surface before functionalization (sample named “DynaEpoxy” in Table 5), but this quantity increases when protein G is conjugated to the surface and even more when a-TC Ab is bound (Table 5), attesting to the correct binding of molecules after each step of conjugation. Taken together, these results indicate that DynaEpoxy was correctly functionalized with a-TC Ab. Unfortunately, the XPS analysis was not informative for PureCubeNHS since the signal related to the nitrogen content of antibody could not be distinguished from the much stronger nitrogen signal of NHS group, already present on the surface of the untreated beads.

#### 3.2.3. Binding/Elution Test Using Antibodies

**Figure 10:**
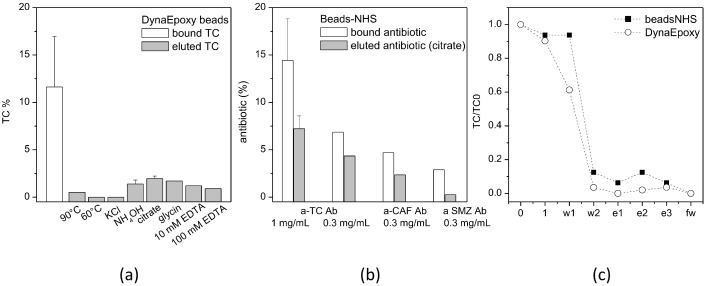
Binding and release of antibiotics from beads functionalized with antibodies. (**a**) Binding to DynaEpoxy beads functionalized with a-TC-Ab through protein G and elution of TC with different conditions. The percentage of TC is referred to the initial amount of antibiotic added to beads, set as 100%. (**b**) Binding of different antibiotics to PureCubeNHS beads functionalized with the respective antibodies and elution with citrate. (**c**) On-chip purification of TC captured by a-TC Ab bound to PureCubeNHS (solid squares) or DynaEpoxy (open circles).

The microbeads functionalized with antibodies were tested for the specific capture and release of their respective antibiotics (Figure 10). Starting from DynaEpoxy beads conjugated with a-TC Ab, the conditions for TC elution were studied in terms of temperature, ionic strength, extreme pH and ion chelators (Figure 10a). All these conditions are expected to perturb the interaction between a-TC Ab and TC, releasing the antibiotic in solution for further analyses. Among the different conditions tested, the elution with citrate, i.e., 100 mM citrate pH 3, gave better TC recovery, and therefore, citrate was selected as the preferred elution buffer for further tests. a-TC Ab was also used to functionalize PureCubeNHS beads to compare the performance of the two types of microbeads in similar conditions (1 mg/mL a-TC Ab, 1 ng/µL TC, elution with citrate; Figure 10a,b). The capture and especially the release of TC was higher when PureCubeNHS was used, resulting then in being more promising for evaluating the performance of the other antibodies. PureCubeNHS was therefore functionalized with the three different antibodies at the same initial concentration, i.e., 0.3 mg/mL (Figure 10b). The same amount of the three antibiotics (i.e., 1 ng/µL) was incubated with the matching antibody-functionalized beads, finding both good binding and good recovery for a-TC Ab and a-CAF Ab, while for a-SMZ Ab binding, and especially elution, lower performance results were observed. These data are indeed in good agreement with the results obtained with the filtration test (Figure 8 and Table 4).

Beside tests in vials, a-TC Ab was selected as case study for setting up the on-chip purification of TC through both types of microbeads (Figure 10c). A solution of 0.1 ng/µL of TC spiked in binding buffer, i.e., the lower amount allowed by regulations, was flowed in the chip filled with the functionalized microbeads, and the collected fractions were quantified by HPLC. Again, the PureCubeNHS beads are able to release slightly more TC than DynaEpoxy. However, these findings clearly indicate a much worse performance, when compared with those obtained with aptamers in similar conditions (Figure 7), suggesting not to proceed with testing the on-chip purification of antibiotics spiked in raw food.

### 3.3. Strategy Based on Small Molecules (Sulfanilic Acid)

The third strategy considered for capturing antibiotics on the functionalized microbeads relies on the binding of small molecules to the beads surface in order to expose suitable chemical groups (Figure 2, strategy c). This strategy was demonstrated as very promising for TC purification by means of microbeads exposing copper ions [20], with the only limit to be valid solely for tetracyclines.

#### 3.3.1. Characterization of Sulfanilic Acid Binding

Here, sulfanilic acid was reacted with DynaEpoxy or PureCubeNHS beads in order to introduce a strong negatively charged group (SO${}_{3}^{-}$), able to capture sulfonamides in acid environment. The correct functionalization was verified for DynaEpoxy treated with sulfanilic acid via XPS analysis (Table 6). Untreated DynaEpoxy does not contain any sulfur, and therefore the presence of S 2p is only due to the reaction of sulfanilic acid. On treated DynaEpoxy beads, indeed, a small but detectable amount of sulfur is found (Table 6).

XPS analysis was performed also on PureCubeNHS beads before and after the treatment with sulfanilic acid, but in this case, no differences were observed, indicating that sulfur, if present, is below the sensitivity of this technique.

#### 3.3.2. Binding/Elution Test

**Figure 11:**
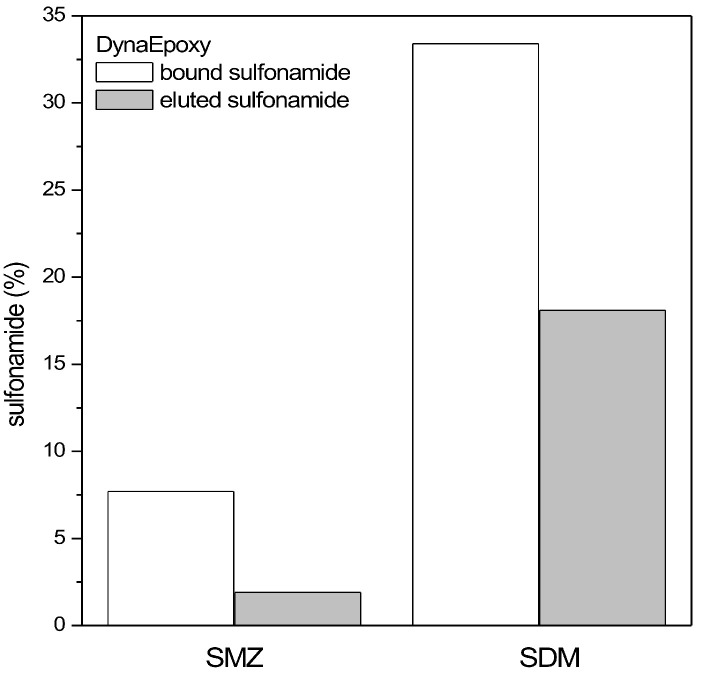
Binding and elution test of sulfonamides from DynaEpoxy beads functionalized with sulfanilic acid. The percentage of sulfonamides is referred to the initial amount of antibiotic present in the reaction.

DynaEpoxy beads functionalized with sulfanilic acid were tested for the binding and elution of two sulfonamides, i.e., SMZ and SDM (Figure 11). The best performance reuslts in terms of both the capture and release of antibiotics were obtained for SDM, while SMZ was bound in a little amount (8%) to the beads and eluted in an even minor amount (2%). The binding test was performed also with PureCubeNHS beads and SMZ, finding an even smaller binding of the antibiotic (4 ± 2%) and no detectable elution. The quite good result obtained using DynaEpoxy beads and SDM, however, requires harsh binding conditions (HCl 0.1 M) as well as quite harsh elution conditions (NaOH 28%/methanol in 35/65 v/v ratio), making this strategy quite difficult for raw food processing. Moreover, these harsh conditions hinder the use of the eluted antibiotic for further analyses without any treatment, making, in this way, the whole idea behind the purification process ineffective.

## 4. Conclusions

The microfluidic purification of antibiotics could be a powerful instrument to detect traces of residual antibiotics in food before they enter the food chain, becoming a great issue for human health and for the environment. Microbeads inserted into a microchamber lead to an increased surface-to-volume ratio of the microdevice. Therefore, if properly functionalized, they have a huge potential in purifying and concentrating also diluted antibiotics for easier quantification. Combining the two technologies, i.e., microfluidic and functionalized microbeads, could offer an improved approach toward an efficient, rapid, automated and easy-to-use analysis. Here, three strategies were evaluated for the functionalization of microbeads and used for binding and eluting antibiotics. Every strategy has its own pros and cons, but taking together all the presented results, the most promising strategy for the on-chip purification of antibiotics seems to be the strategy based on aptamers. Aptamers gave the highest yield in antibiotic capture and, at least one aptamer (i.e., a-TCmu), was able to concentrate almost two times the initial amount of antibiotics. Unfortunately, all the strategies evaluated in this paper showed some weak points when applied to the purification of antibiotics directly from raw food, leaving room for improvement in the interactions between the target and its ligand in complex food matrices, such as milk or honey.

## Figures and Tables

**Table 1:** List of aptamers tested. Aptamers were all DNA based, except for a-TCmu, which was RNA based. They were purchased without modifications or with one of the modifications listed in the last column. Some aptamers were also modified at the 3${}^{\prime }$ end with a fluorescent label (/36-TAMSp/). All modifications are referred to the standard nomenclature of the manufacturer. TC: tetracycline; SDM: sulfadimethoxine; SMZ: sulfamethazine.

Name	Sequence (5${}^{\prime }$-3${}^{\prime }$)	Target	5${}^{\prime }$ Modification
a-TC8	CGG TGG TG	TC	/5AmMC12/; /5ThioMC6-D/
a-TC40	GTT TGT GTA TTA CAG TTA TGT TAC CCT CAT TTT TCT GAA C	TC	/5AmMC12/
a-TC76	CGT ACG GAA TTC GCT AGC CCC CCG GCA GGC CAC GGC TTG GGT TGG TCC CAC TGC GCG TGG ATC CGA GCT CCA CGT G	TC	/5AmMC12/; /5ThioMC6-D/; /5Biosg/
a-TCmu ^1^	GGG CCU AAA ACA UAC CAG AUC GCC ACC CGC GCU UUA AUC UGG AGA GGU GAA GAA UAC GAC CAC CUA GGC UC	TC	/5Biosg/
a-SDM	GAG GGC AAC GAG TGT TTA TAG A	SDM	/5Biosg/; /5ThioMC6-D/
a-SMZ	TTA GCT TAT GCG TTG GCC GGG ATA AGG ATC CAG CCG TTG TAG ATT TGC GTT CTA ACT CTC	SMZ	/5Biosg/; /5ThioMC6-D/

^1^ RNA sequence.

**Table 2:** List of nonsense (NS) aptamers. All modifications are referred to the standard nomenclature of the manufacturer.

Name	Sequence (5${}^{\prime }$-3${}^{\prime }$)	5${}^{\prime }$ Modification
NS-NH${}_{2}$	CCG TCG AGC AGA GTT CCG TCG AGC AGA	/5AmMC12//iSp9/
NS-Thio	CCG TCG AGC AGA GTT CCG TCG AGC AGA	/5ThioMC6-D//iSp9/
NS-b	GT TGG GCA CGT GTT GTC TCT CTG TGT CTC GTG CCC TTC GCT AGG CCC ACA	/5BiotinTEG/

**Table 3:** Parameters obtained from data fitting of the equilibrium filtration test for the interaction of aptamers with TC.

Aptamer	*k* (nM)	λ	${\mathrm{TC}}_{\mathrm{aspecific}}$ (nM)
a-TC40	100	0.53	12.4
a-TC76	$1.4\times 10^{4}$	0	2.9
a-TCmu	64	≃0	3.5

**Table 4:** Parameters obtained from data fitting of the equilibrium filtration test for the interaction of antibodies with their target antibiotics. ND: not determined.

Antibody	*k* (µM)	λ	$L_{\mathrm{aspecific}}$ (nM)
a-TC Ab	1.6	0	33
a-CAF Ab	0.6	0	25
a-SMZ Ab	ND	-	-

**Table 5:** Elemental composition (%) determined by XPS analysis. The error does not exceed the 1–2% of the reported value.

Sample	O (%)	N (%)	C (%)	Si (%)
DynaEpoxy	32.0	3.9	61.3	2.8
DynaEpoxy + pt G	28.3	4.1	65.4	2.2
DynaEpoxy + pt G + EA + a-TC Ab	28.9	5.2	64.4	1.6

**Table 6:** Elemental composition (%) determined by XPS analysis. The error does not exceed the 1–2% of the reported value.

Sample	O (%)	N (%)	C (%)	S (%)
DynaEpoxy	28.7	4.1	67.2	0.0
DynaEpoxy + sulfanilic acid	27.5	4.4	68.0	0.1

## Data Availability

The data presented in this study are available from the corresponding author upon request.

## Supplementary Materials

The following supporting information [29,30,31,32] can be downloaded at: https://www.mdpi.com/article/10.3390/bios13030325/s1. Methods: Fit of the antibiotic-macromolecule titration; Methods: Functionalization of microbeads. Figure S1: FE-SEM analysis of the morphology of all types of microbeads used in this study.

## Abbreviations

The following abbreviations are used in this manuscript:

TC	Tetracycline
CAF	Chloramphenicol
SMZ	Sulfamethazine
SDM	Sulfadimethoxine
EA	Ethanolamine
ptG	Protein G
