# Supplementary material for: Different Strategies for the Microfluidic Purification of Antibiotics from Food: A Comparative Study
Source: Biosensors (Basel). 2023 Feb 27;13(3):325. doi: 10.3390/bios13030325 (PMC10046095; doi:10.3390/bios13030325)
Supplement: Supplementary file 1 [file biosensors-13-00325-s001.zip › biosensors-2213435-supplementary.pdf]

biosensors

MDPI

Supporting Informaiton

Different strategies for the microfluidic purification of antibiotics from food: a comparative study

Lorenzo Lunelli [1,2], Martina Germanis [1,3], Lia Vanzetti [1] and Cristina Potrich [1,2,*]

[1] Fondazione Bruno Kessler, Center for Sensors & Devices, via Sommarive 18, I-38123 Povo (Trento), Italy
[2] Consiglio Nazionale delle Ricerche, Istituto di Biofisica, via alla Cascata 56/C, I-38123 Povo (Trento), Italy
[3] FTH Srl (Femtorays) via Solteri 38, I-38121 Trento, Italy
* Correspondence: cpotrich@fbk.eu

Methods

Fit of the Antibiotic-Macromolecule Titration.

The amount of bound antibiotics present in the solutions prepared as described above was estimated from the saturation parameter $\nu \equiv L/M$, i.e., defined as the moles of bound ligand (antibiotic) per mole of macromolecule (aptamer or antibody), expressed using the following equation from *Cantor and Schimmel Biophysical chemistry* (section 15-3, [30]) as

$$\nu = \frac{B_{max} \cdot L_f}{k + L_f} \quad \text{(S1)}$$

In Equation (S1), k is the site microscopic dissociation constant, L_f the free antibiotic concentration and B_{max} is the number of sites per macromolecule that are able to bind the ligand. B_{max} is in our case fixed to 1 for the aptamers and to 2 for the antibodies. Considering that $L_f = L_0 - L_b$, $L_b = \nu \cdot M$, (where L_0 is the total antibiotic concentration, L_b is the bound antibiotic concentration and M is the total macromolecule concentration), ν can be readily expressed in the function of the known parameters L_0, B_{max}, M and the fitting parameter k.

From this equation, L_b and L_f can be obtained and used to build two different models for the two experiments described in this section.

(a) TC fluorescence:

The fluorescence F of tetracycline in equilibrium conditions can be expressed by means of the following equation:

$$F = F_b \cdot L_b + F_f \cdot L_f \quad \text{(S2)}$$

where F_b and F_f are the molar fluorescence of tetracycline when bound to the aptamer and free in solution, respectively, whose values are obtained as fitting parameters of the model, alongside the equilibrium constant k.

(b) Equilibrium filtration experiments:

The concentration $L_{through}$ of antibiotics present in the lower filter compartment is considered the sum of two contributions: the free antibiotic (that can pass through the filter), plus a partial ($0 \leq \lambda \leq 1$) contribution from the bound antibiotic coming from both the possible disruption of the complex and from a partial leakage of the filter:

$$L_{through} = L_f + \lambda \cdot L_b \quad \text{(S3)}$$

Moreover, in this case, we consider the effects of a possible non-specific adhesion of the ligand on the filter surface, i.e., we define a decreased, effective total antibiotics concentration $L_{eff} \equiv L_0 - L_{aspecfic}$, and we then substitute L_0 with this effective parameter in Equation S1, obtaining from Equation S3 the following Equation S4:

$$L_{through} = L_0 - L_{aspecific} + (\lambda - 1) \cdot \nu \cdot M \quad \text{(S4)}$$

with k, λ, and $L_{aspecific}$ as fitting parameters of the model.

The fitting of experimental data was implemented in Octave v. 6.1.0 [31], with the *optim* package v.1.6.0, using TeXmacs v. 2.1.1 [32] as the graphical front-end.

Functionalization of Microbeads

The microbeads were functionalized by adapting the manufacturer's instructions, as described below.

DynaEpoxy. About 1 mg of lyophilized beads for each experiment was weighted and resuspended in 1 mL of 0.1 M sodium phosphate buffer pH 7.4, vortexed for 30 s and incubated with tilting and rotation for 10 min. After removing the supernatant on a magnet, the same washing step was repeated, and beads were resuspended in 100 µL of 0.1 M sodium phosphate buffer pH 7.4. Then, the selected amino-terminated aptamer was added at the final concentration of 10 µM (besides a-TC8, which was added at 20 µM final concentration), followed by ammonium sulfate at a 1.5 M final concentration. This incubation was performed for 24 h at 400 rpm and at room temperature (a temperature of 60 °C was also tested). Aptamers were added to the beads after a thermal shock to favor the specific binding. The supernatant was collected and measured at the spectrophotometer in order to quantify the bound aptamer (Spectrophotometric analysis, Section 2.4.3). Four more washes with PBS were performed before DynaEpoxy beads were ready for incubation with the antibiotic. DynaEpoxy were also conjugated to specific antibodies with the same protocol as for aptamers (besides the binding temperature, which was set to 37°C) or with an intermediate step of conjugation with protein G (Thermo Scientific; Waltham, MA, USA) in order to better orient the antibodies. Moreover, DynaEpoxy was also modified for the capture of sulfonamides by introducing a sulfone group through the direct conjugation of sulfanilic acid to epoxy groups of the microbeads. Briefly, 1 mg of beads resuspended and washed were incubated with 0.46 M sulfanilic acid in 0.5 M sodium carbonate pH 10 at 1000 rpm and 60°C for 48 h, adapting the protocol described in Hirsch et al. [33].

DynaSA. For each experiment, 100 µL (i.e., 1 mg) of the bead suspension was transferred to a 1.5 mL vial and placed on a magnet, and the supernatant was discarded. After 4 washes with B&W buffer, the biotinilated aptamer was added at the final concentration of 20 µM in B&W buffer and incubated with tilting and rotation for 15 min at room temperature. At the end of incubation, the supernatant was collected for the spectrophotometric quantification (Spectrophotometric analysis, Section 2.4.3), and the beads were washed twice with B&W buffer and then resuspended in a suitable buffer for the antibiotic capture.

MagarNA. For each experiment, 30 µL of beads suspension, i.e., 1X condition, were transferred in a microcentrifuge tube and placed on a magnet, and the supernatant was discarded. The beads were then washed four times with B&W buffer and resuspended in 50 µL of the same buffer. Then, 50 µL of aptamer was added to a final concentration of 10 µM in B&W buffer and incubated with tilting and rotation for 15 min at room temperature. At the end of incubation, the supernatant was collected for the spectrophotometric quantification, and the beads were washed twice with B&W buffer and then resuspended in binding buffer for TC capture.

PureCubeNHS. For each experiment, 30 µL of the beads suspension was transferred in a microcentrifuge tube and placed on a magnet, and the supernatant was discarded. The beads were then washed twice with 1 mL PBS and the specific amino-terminated aptamer was added (after a thermal shock) at a final concentration of 10 µM (besides a-TC8, which was 20 µM). The incubation was performed at room temperature with tilting and rotation for h. After incubation, the supernatant was collected for the spectrophotometric analysis, and the beads were washed once with PBS and 4 times with ultrapure water,

before passivating the unreacted -NHS groups with 1 M ethanolamine. This reaction was performed for 1 h at room temperature with tilting and rotation. After 4 washes with PBS and 2 with ultrapure water, the beads were used for the incubation with antibiotics.

PureCube Maleimide. For each experiment, 50 µL of the bead suspension was transferred in a microcentrifuge tube and placed on a magnet, and the supernatant was discarded. After 3 washes with CBI buffer, the reduced aptamer was added and incubated with tilting and rotation for 2 h at room temperature. Thiolated aptamers were reduced just before use with 10 mM DTT in CBI for 1 h at room temperature and 400 rpm agitation. DTT was then removed with Amicon ® Ultra-0.5 Centrifugal Filter Devices (Merck Life Science S.r.l.; Milan, Italy), and the final concentration of the aptamer was quantified by spectrophotometry (Nanodrop ND-1000 spectrophotometer, Nanodrop Tecnologies, Wilmington, DE, USA). After incubation with the aptamer, the beads were washed first with CBI and then with the buffer suitable for the following incubation with antibiotic.

Morphological Characterization of Microbeads (FE-SEM Analysis)

All microbeads used in this study were analyzed for their morphology by FE-SEM. Microbeads were prepared for performing conjugation experiments but were resuspended in pure water just before being deposited on thermally grown silicon oxide substrates. The substrates were treated with an argon plasma for 1 min at 2 mbar and 10.5 W for improving bead adhesion. A few microliters of beads were deposited on the substrates, dried in air and coated with 10 nm gold (sputter coater EMITECH K575X). Imaging was performed with the PFIB-SEM Helios 5 electron microscope (ThermoScientific), using the charge compensation mode.

Images of microbeads are reported in the figure below (Figure S1).

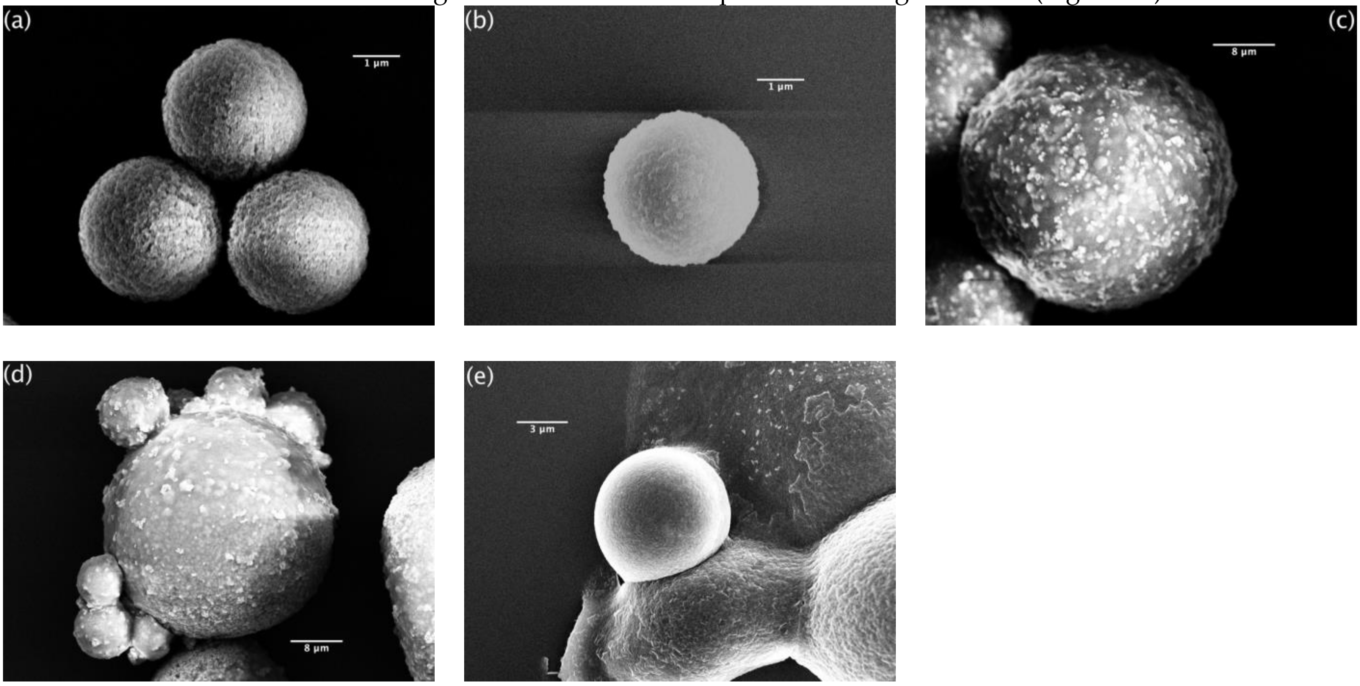

Figure S1. FE-SEM analysis of the morphology of all types of microbeads used in this study: (**a**) DynaEpoxy, (**b**) DynaSA, (**c**) PureCube Maleimide, (**d**) PureCubeNHS, (**e**) MagarNA. Magnifications were, respectively, 60.000×, 60.000×, 8.000×, 7.000× and 16.000×.

References

30. Cantor, C.R.; Schimmel, P.R. *Biophysical chemistry: Part III: the behavior of biological macromolecules*; Macmillan: New York, NY, USA, 1980.
31. Eaton, J.W.; Bateman, D.; Hauberg, S.; Wehbring, R. *GNU Octave version 6.1.0 manual: a high-level interactive language for numerical computation*s; Octave: San Francisco, CA, USA, 2020.
32. van der Hoeven, J. Towards semantic mathematical editing. *J. Symb. Comput.* **2015**, *71*, 1–46.
33. Hirsch, D.B.; Baieli, M.F.; Urtasun, N.; Lázaro-Martínez, J.M.; Glisoni, R.J.; Miranda, M.V.; Cascone, O.; Wolman, F.J. Sulfanilic acid-modified chitosan mini-spheres and their application for lysozyme purification from egg white. *Biotechnol. Prog.* **2018**, *34*, 387–396.
